# Perovskite fluid dynamics

**DOI:** 10.1016/j.fmre.2025.08.007

**Published:** 2025-08-29

**Authors:** Changshun Chen, Yingdong Xia, Yonghua Chen

**Affiliations:** a State Key Laboratory of Flexible Electronics (LoFE) & Institute of Advanced Materials (IAM), School of Flexible Electronics (Future Technologies), Nanjing Tech University, Nanjing 211816, China; b State Key Laboratory of Flexible Electronics (LoFE) & Frontiers Science Center for Flexible Electronics, Institute of Flexible Electronics (IFE), Northwestern Polytechnical University, Xi'an 710072, China

**Keywords:** Perovskite, Fluid dynamics, Phase transition, Film morphology, Stability

## Abstract

The phase transition of perovskite thin films is inherently rapid and highly sensitive to multiple factors. Fluid dynamics has emerged as a powerful framework for elucidating the crystallization and phase transformation processes in perovskites. It governs solute transport, energy dissipation pathways, and mass transfer efficiency, thereby exerting a significant influence on nucleation behavior and crystal growth dynamics. These factors are critical for optimizing film morphology and enhancing device performance in large-area perovskite modules. In this perspective, we first explore the regulatory role of fluid dynamics across distinct phase transition stages and the underlying mechanisms. Then, its practical value in improving film uniformity and optoelectronic performance is examined, with particular emphasis on controlling crystal growth orientation and enhancing structural consistency. Such regulation is considered beneficial for improving device performance and ensuring long-term operational stability under ISOS protocols. Finally, we outline emerging opportunities in solution flow control, crystallization induction, defect passivation, and structural design. These insights offer promising directions for addressing key challenges in stability and scalable fabrication, thereby advancing the development of low-cost, manufacturable perovskite technologies.

## 1 Introduction

Over the past two centuries, the field of fluid dynamics has undergone a profound transformation, evolving from the study of ideal fluids governed by the Euler equations to the inclusion of viscous effects described by the Navier–Stokes equations [1]. This evolution has led to significant advancements across a wide range of fields, including aerospace, energy systems, biomedical engineering, and semiconductor manufacturing. In recent years, fluid dynamic principles have also been increasingly applied to the investigation of phase evolution during perovskite thin film formation, highlighting the critical role in regulating film quality and device performance [2,3].

Fluid dynamics is the branch of physics that describes the motion of fluids and the forces acting on them. It provides the theoretical basis for understanding momentum, mass, and energy transport within liquid or gas systems. In solution-processed materials such as metal halide perovskites, fluid behavior directly affects solute transport, solvent evaporation, and interface evolution. These factors are particularly critical during the crystallization process, where local flow conditions determine nucleation uniformity, crystal growth direction, and phase transformation pathways. As fabrication advances from small-area test films toward large-area perovskite modules, achieving uniform film quality increasingly relies on precise regulation of fluid flow and associated transport processes. Consequently, fluid dynamics offers new possibilities for controlling crystallization kinetics and improving film morphology, enabling scalable coating strategies for achieving high performance and long-term stability in perovskite photovoltaics.

## 2 Fluid dynamics in perovskite phase transitions

To understand the role of fluid dynamics in perovskite thin film formation, Fig. 1 illustrates the underlying physical mechanisms involved, encompassing the stages of nucleation, crystal growth, phase transition, and final film deposition. The upper-right panel summarizes typical aging behaviors of precursor solutions, in which Pb-based complexes play a key role in influencing the uniformity and stability of perovskite colloids. These aging effects can disrupt fluid flow, hinder solvent evaporation, and adversely affect crystal growth and film morphology during phase transformation [4]. During solvent evaporation, complex internal flow patterns develop within the wet film, including Marangoni flow, thermal convection, and mass transfer driven by concentration gradients. These fluid movements significantly affect the migration paths, local accumulation, and spatial distribution of colloidal species. Four representative kinetic models are presented to describe the key stages of perovskite crystallization. Equation (1), which corresponds to the homogeneous nucleation free energy model, explains the energetic competition between supersaturation (C/C₀) and interfacial energy (γ). Equation (2), the heterogeneous nucleation model, introduces the role of substrate wettability via contact angle (θ) in reducing interfacial energy barriers [5]. Equation (3) captures diffusion-limited crystal growth, where the rate depends on the concentration gradient (dC/dx) and the diffusion coefficient (D). Equation (4), the Avrami model, provides a quantitative framework for phase transition kinetics by relating the parameter *n* to nucleation rate and growth dimensionality. During perovskite film formation, crystallization typically proceeds through several stages: initial nucleation, directional crystal growth, and late-stage structural rearrangement. Throughout this process, fluid motion not only facilitates solute transport but also influences interfacial stress distribution, crystal orientation, and overall film quality. Fluid instabilities or uneven solute distribution often cause excessive localized nucleation, misaligned crystal domains, and the formation of irregular film morphologies such as wrinkling, banding, and edge accumulation. In large-area printing techniques such as blade coating and slot-die coating, uncontrolled Marangoni flow or deviations from the Landau-Levich regime can lead to fluctuations in film thickness and spatial inhomogeneity [6]. Overall, fluid dynamics provides a valuable framework for linking phase transformation stages to crystallization kinetics in perovskite systems, forming a theoretical foundation for optimizing film formation in scalable manufacturing.

**Fig. 1:**
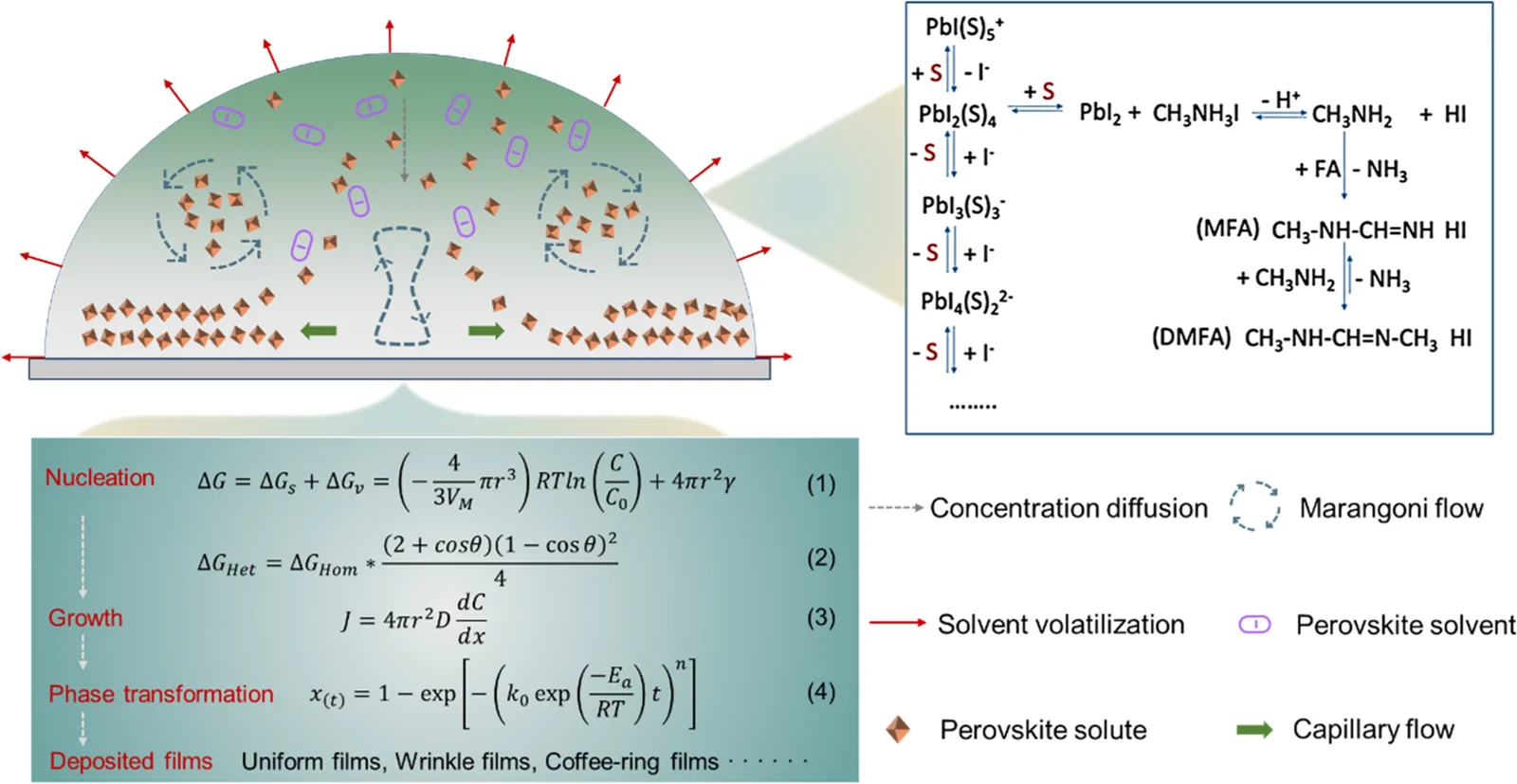
**Fluid dynamics in the crystallization and phase transition of perovskites.** During the deposition process, perovskite fluids experience complex internal flow influenced by Marangoni flow, concentration-driven diffusion, and capillary effects, resulting in various film morphologies, including uniform films, wrinkled films, and coffee-ring patterns. Equation (1) is the kinetics of perovskite crystallization, which could be understood through the Gibbs free energy change (*ΔG*) for nucleation. Equation (2) is the free energy required for heterogeneous nucleations (*ΔG_Het_*), which could be expressed as a function of the free energy required for homogeneous nucleations (*ΔG_Hom_*) and the contact angle. The diffusion-growth model of perovskite in the early stage can be described by Fick's first law (Equation [3]). Equation (4), derived from the Johnson–Mehl–Avrami (JMA) model, describes the phase transformation process of perovskite.

## 3 Application of fluid dynamics in perovskite films

Perovskite modules are considered a key pathway to achieving high efficiency, low cost, and scalability in perovskite photovoltaics. However, significant challenges persist in achieving device stability and film uniformity, especially over large-area perovskite thin films. In this context, fluid dynamics has become a powerful theoretical tool for regulating large-area crystallization behavior and improving module performance [7]. During perovskite thin film formation, fluid behaviors such as fluid motion, interfacial wetting, and solvent evaporation have been shown to significantly influence nucleation distribution, orientation uniformity, interface-induced crystallization, and overall film morphology. When scaling up film dimensions, fluid dynamics become even more critical, as non-uniform flow and solvent evaporation may lead to thickness variations, crystallization gradients, and stress-induced defects. Recent strategies such as tuning Marangoni convection, optimizing airflow, and homogenizing solute distributions have been proposed to address these issues and improve the uniformity and scalability of large-area films [8,9]. These factors directly affect the film quality and defect density of perovskite thin films, thereby influencing the optoelectronic performance and long-term operational stability of large-area perovskite modules.

Regulating fluid dynamics is essential for optimizing the crystallization process of perovskite materials. Fig. 2 outlines various strategies that highlight the critical role of fluid dynamics in scalable module fabrication. In this context, solvent evaporation and fluid motion are crucial factors in determining film uniformity and crystallization quality [10]. By adjusting the evaporation rate, solvent composition, and interfacial tension gradients, solute accumulation and non-uniform crystallization can be effectively suppressed. Recent studies have demonstrated that introducing methanol (MeOH) as a fluid-balancing additive effectively controls the direction of Marangoni convection, suppressing the random motion of colloidal species within wet perovskite thin films and significantly enhancing device efficiency in large-area modules [3]. Meanwhile, solvent evaporation and unbalanced fluid flow can also act as sources of internal stress during film formation. Rapid shrinkage, shear gradients, and flow-driven solute redistribution may induce localized stress accumulation, which affects nucleation behavior, grain orientation, and ultimately film integrity. Recent findings have highlighted that careful control of flow conditions, such as laminar flow profiles or fluid homogenization, can mitigate stress buildup and promote more uniform crystallization, particularly in scalable coating processes [9]. Additionally, the formation and conversion of intermediate phases are crucial for achieving ordered crystal packing and orientational consistency. Fluid dynamics can influence the solvent-solute dissociation process, compositional uniformity, and flow-field stability, which together impact the transformation rate and structural order during phase transitions. Several approaches, including amine vapor exposure and vacuum-driven precrystallization, have been shown to effectively guide these processes [11]. In parallel, reviews on perovskite single-crystal have provided valuable insights into nucleation kinetics and solute-solvent interactions, benefiting the understanding of crystallization mechanisms [12,13]. Furthermore, the introduction of interfacial materials and passivating molecules has emerged as a promising approach to regulating fluid dynamics in perovskite processes. By modulating the wetting behavior between the precursor solution and substrate materials, the spreading process and flow characteristics of the perovskite solution can be significantly influenced, thereby controlling the nucleation process and crystallization orientation [14]. Additionally, the solubility, coordination properties, and interfacial affinity of passivating materials determine the diffusion behavior and enrichment regions of perovskite colloids, directly affecting the passivation of defects in perovskite thin films. The fluid reorganization processes induced by these materials provide new strategies for improving the quality of perovskite thin films and enhancing module stability.

**Fig. 2:**
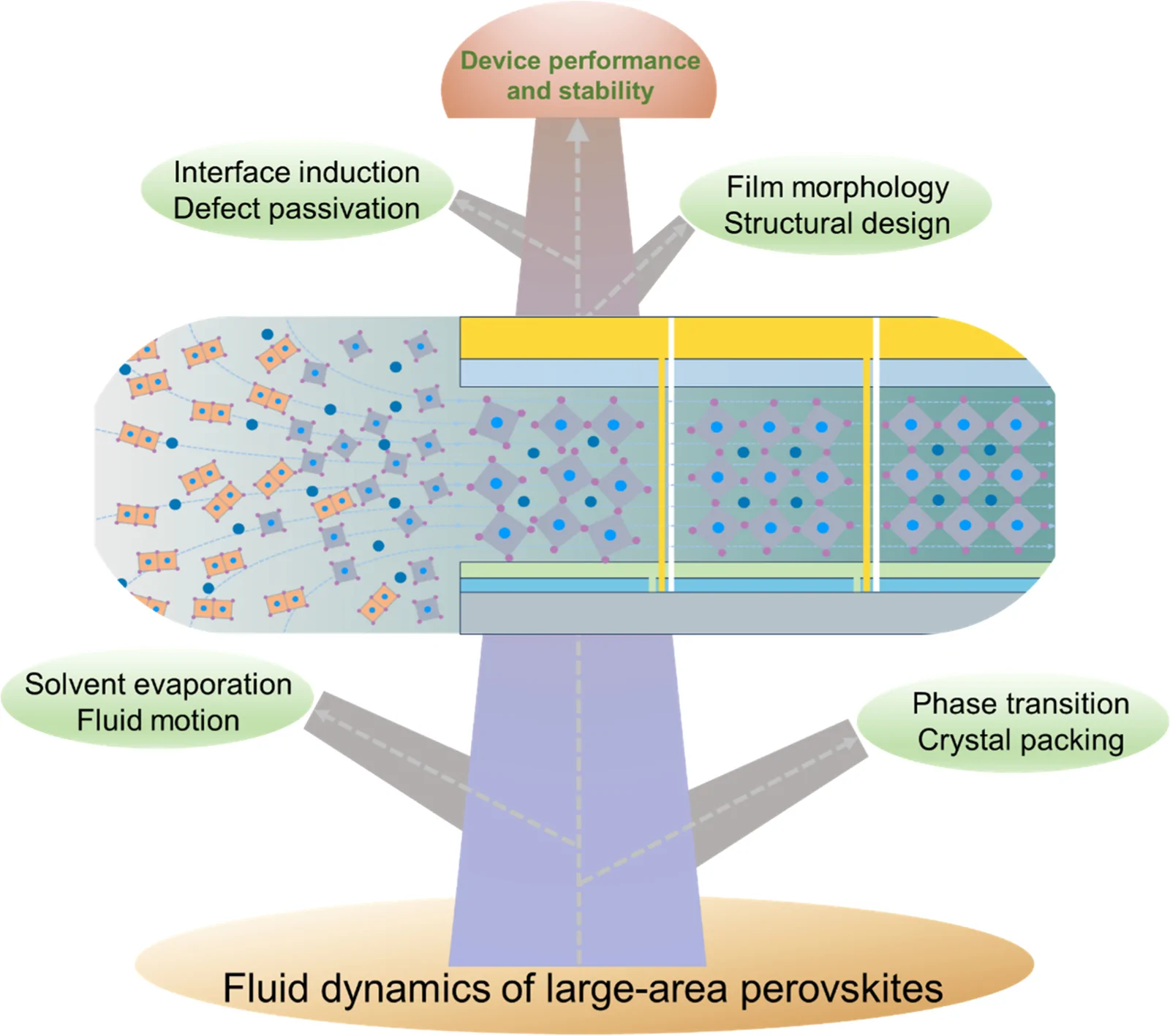
**Fluid dynamics in controlling large-area perovskite thin films and modules.** Fluid dynamics influences solvent evaporation, fluid motion, phase transition, crystal packing, interface induction and defect passivation during film formation. These factors collectively determine the morphology and uniformity of perovskite thin films, ultimately impacting device performance and operational stability in large-area perovskite modules.

## 4 Summary and challenges

Overall, this perspective underscores the critical role of fluid dynamics in regulating phase transformation, crystallization, and structural uniformity in large-area perovskite photovoltaics. Fluid dynamics governs kinetic processes and morphological evolution, offering a coherent framework for understanding film formation and ensuring the stability of perovskite modules. By bridging microscale regulation with scalable module fabrication, this approach provides both conceptual insight and practical guidance. Further exploration of fluid-driven strategies is expected to advance the development of reliable and manufacturable perovskite photovoltaic technology.

Despite recent progress, several challenges persist. Current studies remain fragmented, with limited systematic understanding of how flow velocity, shear gradients, and interfacial dynamics affect nucleation and crystal orientation. Theoretical predictions are often difficult to apply in practice due to complex solvent evaporation, colloidal behavior, and varying rheological properties. Most models lack experimental validation and fail to capture the multiscale nature of the process. Furthermore, the coupling between flow, stress evolution, and interfacial energetics remains poorly understood. Addressing these issues is critical for advancing predictive control and enabling scalable, high-quality perovskite film fabrication.

## CRediT authorship contribution statement

**Changshun Chen:** Writing – original draft, Methodology, Formal analysis, Data curation. **Yingdong Xia:** Writing – review & editing, Writing – original draft, Supervision, Methodology, Investigation, Funding acquisition, Conceptualization. **Yonghua Chen:** Writing – review & editing, Supervision, Project administration, Funding acquisition, Conceptualization.

## Declaration of competing interest

The authors declare that they have no conflicts of interest in this work.
